# A real-world feasibility study of the PLAYshop: a brief intervention to facilitate parent engagement in developing their child’s physical literacy

**DOI:** 10.1186/s40814-021-00849-5

**Published:** 2021-05-26

**Authors:** Cassandra Lane, Valerie Carson, Kayla Morton, Kendra Reno, Chris Wright, Madison Predy, Patti-Jean Naylor

**Affiliations:** 1 School of Medicine and Public Health, The University of Newcastle, Newcastle, NSW Australia; 2 Hunter New England Population Health, Hunter New England Area Health Service, Newcastle, NSW Australia; 3 Faculty of Kinesiology, Sport, and Recreation, University of Alberta, Edmonton, Alberta Canada; 4 School of Exercise Science, Physical and Health Education, University of Victoria, Mackinnon 120, PO Box 1700, STN CSC, Victoria, BC V8W 2Y2 Canada; 5 Pacific Institute for Sport Excellence, Victoria, BC Canada

**Keywords:** Physical literacy, Parent, Child, Fundamental movement skills

## Abstract

**Background:**

Development of physical literacy, defined as “the motivation, confidence, physical competence, knowledge and understanding to value and take responsibility for engagement in physical activities for life,” can support children’s physically active behaviors and consequent health benefits. Little research has explored interventions to improve children’s physical literacy, although substantive evidence shows parents play a key role in children’s physically active behaviors and development of fundamental movement skills. The purpose of this study was to explore the feasibility of a novel, physical literacy program (the PLAYshop) designed to build parents’ self-efficacy to support their child’s physical literacy.

**Methods:**

A non-randomized, one-arm concurrent nested design was used. Thirty-five parents of young children (3–8 years of age) attended a 75-min workshop inclusive of interactive activities, educational messages, and the provision of resources focused on core physical literacy concepts. Pre- and post-workshop surveys used quantitative measures to assess parents’ satisfaction, knowledge, confidence, and intention to adopt practices. Follow-up interviews qualitatively explored the implementation experiences of both parents and facilitators. Paired *t* tests and thematic analysis were undertaken.

**Results:**

Of the 33 eligible parents, 23 completed both pre- and post-workshop surveys. Follow-up interviews were completed with 11 parents and four workshop facilitators. Parents’ self-reported knowledge and confidence to support their child’s physical literacy development significantly increased after PLAYshop participation. The majority of parents were satisfied with the workshop and motivated to apply learnings at home with their child. Workshop facilitators identified seven workshop strengths (e.g., workshop champions and skilled facilitators) and four challenges (e.g., recruitment and unfavorable spaces). Limitations include the lack of control group and recruitment challenges.

**Conclusions:**

The PLAYshop was perceived positively by parents and facilitators and appeared to improve parent self-efficacy and intention to promote physical literacy with their child. Recruitment and attendance were key implementation challenges. The findings from this real-world study support the preliminary feasibility of the PLAYshop intervention and highlight areas to improve the intervention and recruitment prior to efficacy testing in a more rigorous trial format.

## Key messages regarding feasibility

- ***What uncertainties existed regarding the feasibility****?* The feasibility of a potentially scalable childhood physical literacy workshop intervention that targets parents (specifically to enhance their knowledge and self-efficacy, and to increase purposeful play to enhance their child’s physical literacy) is currently unknown. In addition, the implementation feasibility in terms of recruitment and delivery is unknown.
- ***What are the key feasibility findings?*** Thirty-five parents were recruited to attend 1 of 6 parent workshops and 23 completed all evaluation components. The PLAYshop was acceptable to parents with 95.4% and 95.5% satisfied or extremely satisfied with the workshop content and delivery, respectively. Nearly all parents were highly motivated to implement activities after the workshop and significant improvements in knowledge, confidence, and self-reported practices resulted. Recruitment and retention of parents for follow-up measures need to be addressed to ensure the success of a full trial.
- ***What are the implications of the feasibility findings for the design of the main study?*** The intervention was well received by parents. Parents require more support after the workshop. Implementation may be supported with an additional workshop leader. Future trials should expand recruitment efforts and recruit parents directly.

## Background

Physical activity is critically important to the development of young children as it improves cognitive and motor skill development, psychosocial health, social connectedness, and cardiometabolic health and reduces adiposity [10, 34] and risk of chronic disease in adulthood [11]. Unfortunately the prevalence of children participating in sufficient levels of physical activity is low internationally [1]. In response, researchers have developed and tested the effectiveness of numerous childhood physical activity interventions [22]. Amidst some success, their impact on children’s levels of physical activity appears limited [23] and several research gaps remain.

Physical literacy offers a relatively new and promising approach for childhood physical activity interventions [9]. It is defined by the International Physical Literacy Association as “the motivation, confidence, physical competence, knowledge and understanding to value and take responsibility for engagement in physical activities for life” [18]. The novelty of physical literacy lies in the equal importance assigned to four key domains: affective (motivation and confidence), cognitive (knowledge and understanding), social (relationships and social networks), and physical capabilities (e.g., motor skill competence and fundamental movement skills [FMS]) [13, 30]. Physical literacy is an antecedent to improve and maintain physical activity participation and consequent health benefits [9, 13], thus it is particularly important to begin development early in the life course [32]. Parents’ meaningful engagement in this physical literacy journey is considered critical [32] due to their influence on their child’s physical activity-related behaviors; supported by systematic review evidence.

In a 2016 systematic review of family-based physical activity interventions, 31 of the 47 included studies demonstrated a significant positive effect on children’s physical activity levels [8]. More specifically, a systematic review of the determinants of physical activity in children aged 0–6 years found that parent monitoring was the only factor consistently associated with children’s physical activity [16, 17]. Lastly, a synthesis of results from 39 high-quality reviews [22] provided strong evidence that parents play a key role in promoting child physical activity across various community settings. Rhodes and colleagues [28] further explored this topic in a recent systematic review of the correlates of parental support on child physical activity. Out of the twenty correlates identified in 19 unique data sets, co-participation, logistical support, and encouragement were the most common elements of interventions with a positive effect on children’s physical activity levels.

Although these previous systematic reviews did not report on physical literacy, many of the included studies examined parent-focused interventions that emphasized FMS—a key part of physical literacy [5, 36] often used synonymously [13]. For example, the 3-month Healthy Dads Healthy Kids (HDHK) program adopted FMS as the exclusive focus for one of the four, 75-min father/child practical sessions [25, 26]. Similarly, the 8-week Dads and Daughters Exercising and Empowered (DADEE) program [27] and the mother-daughter MADE4Life program [3] each devoted at least one practical session to FMS. FMS was an explicit outcome measure within only one intervention trial: a 2018 randomized controlled trial (RCT) of DADEE that found significant improvements in daughters’ FMS proficiency [27]. FMS competence was objectively assessed via scores of daughters’ performance in six object control skills (kicking, catching, striking a stationary ball, stationary dribble, overhand throw, and underhand throw). Compared to controls, daughters in the intervention had significantly higher object control scores post-intervention (*p*<.001) that were sustained at 9-month follow-up (*p* ≤ .002).

Positive findings for physical activity-related outcomes were found in trials of all interventions: fathers in DADEE significantly improved in physical activity parenting practices compared to controls [27]; children in HDHK significantly increased in level of physical activity compared to controls [25, 26]; and mothers in MADE4Life reported positive behavior change as a result of the program [3]. Further, all of these programs appeared feasible, with high attendance and ratings of program satisfaction made by fathers in HDHK [25, 26] and DADEE [27], as well as high ratings of program quality and session content made by mothers in MADE4Life [3].

These parent-focused interventions inclusive of FMS appear to have a meaningful impact on children’s physical activity. However, they required a significant time commitment from parents (multiple sessions over the course of 2–3 months) and were resource-intensive. A simplified intervention may be more accessible for time-restricted parents and be more amenable for scale-up [35]. It is also unclear whether the aforementioned interventions had any impact on parent self-efficacy—an important component of behavior change [2] also linked to children’s physical activity levels [31].

Increasing parent self-efficacy (knowledge, confidence, and capacity) to carry out activities that promote physical literacy may provide children with a foundation for development in this area. No research to date has explored a brief parent-focused physical literacy program as an intervention option. In light of this evidence gap, we developed a novel theory-based program (the PLAYshop) that aimed to build parents’ self-efficacy in assisting their child to develop physical literacy and acquire physical activity through play. The PLAYshop was originally designed by a lead member of the research team in partnership with a representative from a community physical literacy agency (Pacific Institute for Sport Excellence; PISE). It was first tested in two community settings to explore the approach (core physical literacy content, theoretically driven behavior change, and adult education techniques) and feasibility of the format (recruitment, delivery model, etc.). The intervention was then refined for preliminary evaluation in a research context. It was designed with the intent to be scaled up if proven effective within a fully powered efficacy trial, as per WHO recommendations [35].

Before investment in a definitive efficacy and/or effectiveness trial of the PLAYshop, it is important to determine whether the intervention is feasible—that is, “whether the future trial can be done, should be done, and, if so, how” [14]. The overarching goal of the study presented in this paper was to explore the preliminary feasibility of the PLAYshop via assessing its limited efficacy (does the intervention lead to outcomes that are moving in the intended direction) and acceptability (the reaction of those involved with the intervention) [7]. The specific objectives used to achieve this goal were as follows:

1. To assess parents’ knowledge and confidence relating to key physical literacy constructs after participating in the PLAYshop;
2. To explore parents’ experiences and perspectives including their satisfaction with, and perceived usefulness of, the program;
3. To explore program delivery and potential areas of improvement from the perspective of facilitators.

## Methods

### Study design

Six physical literacy workshops (the PLAYshop) were delivered at different times in schools (*n*=4) and sports clubs (*n*=2) within two Canadian jurisdictions. This non-randomized study used a one-arm, concurrent nested approach with quantitative data collected through pre- and post-workshop surveys and qualitative data collected through follow-up telephone interviews of parents and PLAYshop facilitators. Human research ethics approval for the study was obtained from the University of Victoria (#16-444) and University of Alberta (#00093764). As per recommendations [19], the following study methods are reported in accordance with the applicable items from the CONSORT statement for pilot and feasibility trials [14].

### Participants and recruitment

The sampling pool consisted of approximately 1500 families of younger elementary aged-children (estimated based on sizes of school classes and number of sport club members). No sample size calculations were undertaken as the aim of this study was to assess the feasibility prior to a future trial [14]. Parents (fathers and/or mothers) or guardians of young children (ideally 3–8 years of age) were invited to participate in the PLAYshop using e-flyers distributed through the community networks of the schools and local sports clubs. E-flyers provided contact information of the research team responsible for recruitment, workshop scheduling, and evaluation. Parents who responded to recruitment materials were invited to attend a scheduled workshop and siblings of any age were welcome to join and be involved. All parents that showed up on the delivery day were provided with a brief description of the study and invited to participate in the evaluation component. Interested parents were asked to provide written informed consent. Data were obtained from only one parent per family. For example, mothers and fathers of the same family were both welcome to participate in a workshop; however, only one was asked to complete evaluation assessments. Facilitators (that were also members of the study team) were asked if they were interested in participating in an interview about workshop implementation after all scheduled workshops were completed and if so were asked to provide written informed consent in-person or via email. No personal data was collected from the facilitators.

### Intervention

The PLAYshop involved one face-to-face 75-min group workshop and the provision of educational materials. Workshops were delivered to a maximum of 30 parents in community settings by the lead researcher and/or a trained graduate student (each of whom held degrees in physical and health education). The lead researcher had experience in the development of physical literacy and physical activity habits, community-based health promotion, and adult education techniques. The graduate student workshop facilitator was trained by the lead researcher, had completed a 2-h physical literacy education session, and was included in an embedded professional development workshop training approach prior to delivering a full workshop. Facilitator training included first workshop observation, then incremental responsibility (delivering portions of the workshop under the supervision of the lead researcher), and lastly the delivery of significant worskhop portions under supervision. Workshop format and content were guided by the workshop template that was developed by the lead researcher in collaboration with community physical literacy experts (PISE) using evidence-based behavior change techniques [24], adult education and training practices, as well as recommendations and material from international experts in physical literacy development [32, 33].

The aim of the PLAYshop program was threefold: (i) to enhance parents’ understanding of physical activity and physical literacy and their role in facilitating it, (ii) to expose parents to a number of activities and resources that could help them support the development of a wide range of movement skills and increase physical activity, and (iii) to increase parents’ confidence in facilitating playful activities by engaging them in the activities (experiential learning) and providing key messages and modeling approaches that align with, and promote the development of, physical literacy (competence, motivation, confidence, and valuing physical activity).

We employed a systematic process of program design based on Bandura’s social cognitive theory (SCT) [2] and the Behavior Change Wheel (BCW; a synthesis of 19 behavior change frameworks) [24]. Several constructs of SCT (e.g., observational learning, reinforcement, and intentions) were targeted throughout intervention design, as well as development of program components and data collection tools. Our primary focus was self-efficacy—a construct unique to SCT that aligned with our aim to improve parent’s knowledge and confidence to perform target behaviors.

**Fig. 1:**
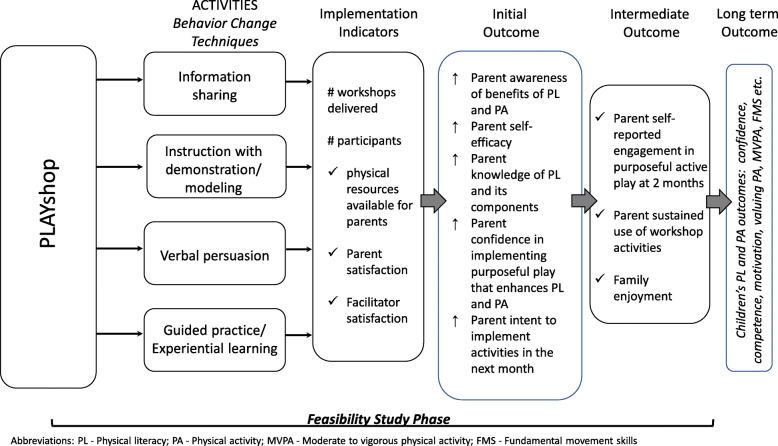
PLAYshop program logic model and components included within the feasibility study phase

The BCW was employed to identify specific strategies to support parents to adopt target behaviors to facilitate cross-jurisdiction knowledge exchange. First, we identified possible barriers and/or enablers to parent’s purposeful play with their child via reviews of the literature and discourse with parents during the development workshops and among the research team. Examples of identified factors that may influence parents to adopt target behaviors include time [16, 17, 21], available resources [16, 17], and confidence [31]. We then classified each factor according to the COM-B model as either capability, opportunity, or motivation. A program logic model is provided in Fig. 1, and the behavior change techniques to address the identified barriers and enablers are detailed in Table 1.

**Table 1:** Description of strategies mapped to the relevant COM-B factors and behavior change techniques

Implementation strategy	Intervention function	Barriers and enablers addressed (COM-B)	Behavior change technique employed	Detailed description
**1. Conduct educational training**	-Education -Training -Modelling -Enablement -Persuasion	Parent knowledge and confidence (psychological capability)	-Information about physical literacy and positive outcomes for the child -Instruction on how to perform the behavior(s) -Instruction on how to perform the behavior(s) using common household items -Demonstration of the behavior(s) -Practice of the behavior(s) -Problem solving -Identification of self (parent) as a role model to the child -Verbal persuasion about capability -Principles of and ideas for modifications to support the parent in meeting the child’s needs in terms of current ability and motivations	A 75-min workshop for parents, delivered in an accessible community site (e.g., school, sport club, or recreation center) by a facilitator with a background in physical literacy. Parents are introduced to the core concepts of physical literacy (motivation, competence, confidence and valuing physical activity) via education, group discussion, and active participation in FMS-based activities. Parents are provided with modifications to perform activities “at home.”
		Parent perceived ability to implement change (physical capability)		
		Lack of available resources and/or time to engage in purposeful play with the child (physical opportunity)		
		Lack of prioritizing child’s physical literacy (reflective motivation)		
**2. Distribute educational resources**	-Education -Enablement	Parent knowledge and abilities (physical and psychological capability)	-Information about physical literacy and positive outcomes for the child -Problem solving -Messages about addressing multiple developmental goals through physical play like numeracy and literacy through singing and counting	Several resources are provided to parents at the workshop conclusion: the Canadian 24-h Movement Guidelines for Children and Youth, cards with various activity ideas, and a one-page physical literacy information handout.
		Lack of available resources and/or time to engage in purposeful play with the child (physical opportunity)		

### Data collection

#### Quantitative data

Pre- and post-workshop surveys of parents collected information about demographics and key study outcomes relating to study objectives 1 and 2. Surveys were developed for the study by the research team and guided by SCT [2]. The outcome measures used to assess study objective 1 were (a) parent knowledge of physical literacy and its key components (physical literacy; locomotor skills, manipulative skills, balance and stability, facilitating physical activities) and (b) parent confidence in promoting physical literacy (providing opportunities for exploration and free play, adapting activities for child’s age/ability, creating a home environment that encourages physical activity, and limiting sedentary behaviors). Instrument sub-scales were adapted from the Activity Support Scale for Multiple Groups (ACTS-MG) [12] and the ParticipACTION Family Physical Activity Questionnaire (Ryan E [29].). Questions used 5-point Likert scale items to measure both parent knowledge (1= no knowledge to 5= a lot of knowledge) and parent confidence (1= no confidence to 5 = a lot of confidence). Parents completed the pre-workshop survey by pen and paper during a 5–10-min period allocated at the start of the workshop. During this time, children and parents that did not complete a survey were offered the opportunity to engage in active games led by the facilitator.

The post-workshop survey included an additional section on parents’ workshop experience (study objective 2) with questions relating to parents’ satisfaction with workshop delivery, whether the content was new to them, and the usefulness of training. This survey was emailed to parents 1 day after their attendance in a workshop with a request to return a completed version via email as soon as possible (with follow-up reminders).

#### Qualitative data

Interviews of parents and facilitators were used to address study objectives 2 and 3, respectively. A member of the research team conducted 10–15-min semi-structured follow-up interviews of parents approximately 2 months after workshop delivery (follow-up). This time period was chosen to provide parents sufficient time to implement a number of workshop activities with their child and to determine the level of implementation over the short term. Parent interviews were composed of open-ended questions to assess the amenability with workshop teachings, the applicability of workshop content, and the ease and/or barriers to implementing learnings with their child/children (study objective 2). A researcher independent of the PLAYshop conducted 10–15-min semi-structured interviews with facilitators within 6 months of the completion of all scheduled workshops. Facilitator interviews used open-ended questions to assess the enablers and barriers to workshop implementation, as well as explore possible modifications to enhance workshop efficacy and useful components that should remain unchanged (study objective 3).

### Data analysis

#### Quantitative data

SPSS Version 21.0 was used to analyze all quantitative data. Demographics and descriptive statistics described the population and baseline levels of knowledge, confidence, and motivation. Paired *t* tests were conducted to calculate the mean changes in parents’ physical literacy knowledge and confidence from baseline to follow-up and corresponding 95% confidence intervals. Statistical significance was defined as *p*<0.05. Measures of frequency (expressed as valid percentages) were calculated to analyze parents’ satisfaction with workshop content and delivery and post-workshop intent to participate in physical activities with their children.

#### Qualitative data

Qualitative data were analyzed following the principles of framework analysis detailed by Gale and colleagues [15]. Two researchers generated themes for parent and facilitator interviews using the following steps: (1) co-coding a subset of transcripts, (2) generating a flexible coding framework, (3) applying this framework to subsequent transcripts, (4) assigning codes to categories, and (5) developing these into themes and sub-themes. The research pair discussed and reached agreeance on any variability that arose during this process. Where consensus could not be achieved, a third member of the research team assisted in final decisions.

## Results

### Sample

Thirty-five parents participated in a workshop. There were two cases in which both parents attended the workshop therefore only one parent from each family was asked to participate in the study evaluation component. All 33 eligible parents provided consent and completed the pre-workshop survey (mean age=38.45 years; 24.2% male, 75.8% female); however, 10 did not return the emailed post-workshop survey. Only data from the 23 parents who completed surveys at both time points were included for analysis. Eleven parents also completed the 2-month follow-up telephone interview; the remainder either declined to participate or was unreachable by phone. The average number of children per household was 1.85. All four PLAYshop facilitators provided consent and completed the facilitator interview.

### Objective 1—Parents’ knowledge and confidence

**Fig. 2:**
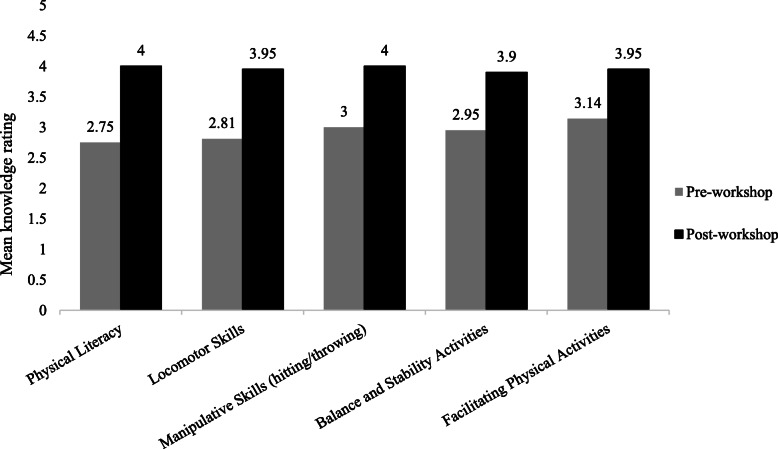
Parents’ self-reported level of physical literacy knowledge pre- and post-workshop

Valid quantitative data for comparison were obtained from pre- and post-workshop surveys completed by 23 parents. Paired *t* test analysis showed a significant increase across all measures of parents’ self-reported knowledge from pre- to post-workshop (*p*≤0.02). Please see Fig. 2. Parents’ mean level of knowledge significantly improved for physical literacy (−1.25 [SD=1.42], *t*(11)=−3.05, *p*=0.011, 95% CI=−2.15 to −0.35), locomotor skills (−1.14, [SD=0.96], *t*(20)=−5.44, *p*=0.000, 95% CI=−1.58 to −0.70), manipulative skills (e.g., catching, striking, kicking, hitting, throwing) (−1.0 [SD=1.21], *t*(19)=−3.68, *p*=0.002, 95% CI=−1.57 to −0.43), balance and stability (−0.95 [SD=1.16], *t*(20)=−3.76, *p*=0.001, 95% CI=−1.48 to −0.42), and facilitating physical activities (−0.81 [SD=1.21], *t*(20)=−3.07, *p*=0.02, 95% CI=−1.36 to −0.26).

**Fig. 3:**
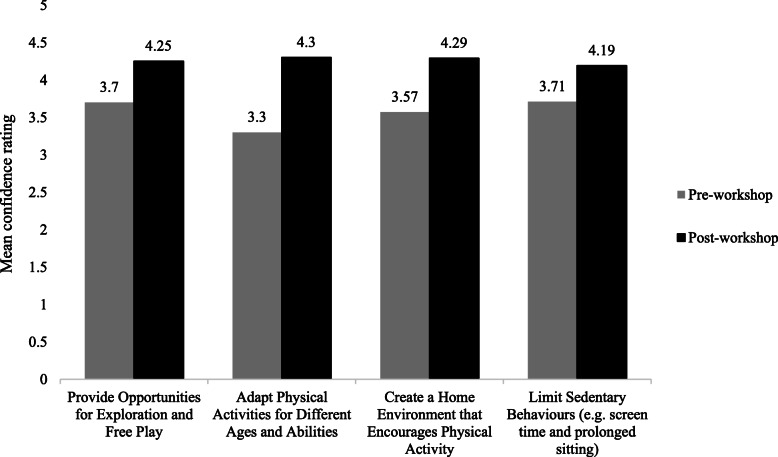
Parents’ self-reported level of confidence to carry out key PLAYshop teachings pre- and post-workshop

Paired *t* test analysis also showed a significant increase across all measures of parents’ self-reported level of confidence for initiating and implementing physical literacy activities with their children from pre- to post-workshop (*p*≤0.009). Please see Fig. 3. Significant improvements were found for parents’ mean level of confidence to provide their child with opportunities for exploration and free play −0.55 [SD=0.76], *t*(19)= −3.24, *p*=0.004, 95% CI=−0.91 to −0.20), to adapt physical activities for different ages and abilities (−1.00 [SD=0.86], *t*(19)= −5.21, *p*=0.000, 95% CI=−1.40 to −0.60), to create a home environment that encourages physical activity (−0.71 [SD=0.72], *t*(20)= −4.56, *p*=0.000, 95% CI=−1.04 to −0.39), and to limit sedentary behaviors such as screen time and prolonged sitting (−0.48 [SD=0.75], *t*(20)= −2.91, *p*=0.009, 95% CI=−0.82 to −0.14).

### Objective 2—Parents’ experiences

Of the 23 parents who completed post-workshop surveys, the majority reported that they were satisfied or extremely satisfied with the workshop content (95.4%) and delivery (95.5%), with no parent reporting anything less than somewhat satisfied (range 3–5). Most parents also found the workshop training very to extremely useful (81.8%). The workshop content was reported as “somewhat new” by 49% of parents and “very new” by 28.6%. Most parents (95.6%) agreed or strongly agreed that they were motivated to do physical activity with their children within 2 weeks of the workshop delivery, and 73.9% disagreed or strongly disagreed that performing physical activities with their child/children within 2 weeks of the workshop would be difficult.

Thematic analysis of the follow-up semi-structured interviews of parents (*n*=11) identified five key themes in relation to implementing the physical literacy activities and concepts (Table 2). Sub-themes are also displayed with illustrative parent quotes. Prominent facilitators were the ease of access/simplicity of activities introduced in the PLAYshop and the enthusiasm of children. Another prominent theme was life barriers getting in the way (i.e., time, routine, and motivation). Parents further suggested that having children of varying developmental stages and receding memories of workshop content over time may pose as an obstacle to using lessons from the PLAYshop.

**Table 2:** Parent feedback in relation to workshop experience and motivation

Theme	Sub-theme	Supporting Quotes
**Simple to use**	o Simple props/supplies o Use what’s available o Accessible	- “Simple and straightforward” - “didn’t require a lot of equipment” - “access is so easy”
**Life gets in the way**	o Time o Motivation o Implementing a routine	- “Setting aside specific time. Scheduling is always hectic” - “Nothing other than my laziness” - “Time probably number 1”
**Kids are interested**	o Skills versus outcomes focused	- “Not make it about how far but the skills level of it” - “more about technique rather than distance” - “Interested, he wants to do it” - “See their excitement and get excited about it”
**Children differ**	o Sibling development stages	- “I usually have both kids at the same time. Difference between kids”
**We need reminding**	o Information fades o No reminders	- “Hard to recall everything. Maybe a follow-up booklet or an outline of theories for activities” - “forget activities”

### Objective 3—Facilitator feedback

Thematic analysis of the semi-structured interviews of workshop facilitators generated two major categories—one relating to strengths and successes (see Table 3) and one relating to challenges and areas for improvement (see Table 4). Each major category was divided into seven themes and four themes, respectively, with some themes fitting within both. For instance, the presence of a champion assisted in the success of some workshops while the absence of a champion appeared as a key challenge for others. Similarly, children attending the workshops served as both a challenge (occasional distraction) and strength (assisted with experiential learning and recruitment), and space for movement also served as either a challenge (inadequate space) or strength (ample space). Facilitator expertise, workshop content, and parents’ positive response to the workshop all emerged as workshop strengths while the need to support parents after the workshop emerged as an area for improvement.

**Table 3:** Workshop strengths and successes from the perspective of workshop facilitators

Workshop strengths and successes	
**Theme**	**Quotes**
**Having a parent or teacher champion**	F1: The 2 schools where we had the most effective recruitment, we had a super-engaged parent that did all the recruiting for us.
	F2: So when people—so for one school that we did [the workshop] in we had a parent advocate in that school who wanted to bring it in and so that really helped because then the turn out for that [workshop] was much better …
**Children attending workshops**	F4: I think it’s important to keep- to have at least part of [the workshop] with their kids. Like get [parents] involved with their own kids right off the bat … . that eliminates big barriers too …then parents don't have to worry about getting child care or anything like that.
	F1: because we got feedback from parents in one of the schools where they weren’t sure if they could bring their children ….They felt [not including kids] reduced attendance …..
	F2: Umm, so we did allow people to bring their kids, which was good because we had enough facilitators there to break off into 2 groups… and then we would come back as a big group. So I think that was … helpful and then we could see how parents are actually interacting with the kids and they can try things right then and there.
	F1: So, you need to be able to riff- on the day you need to be able to riff a bit. Oh and here’s an extension, because sometimes a family comes and they actually have an 8-year old there and a 5-year old, and the activity for the 5-year old and the 8-year old are different. And you need to be able to demonstrate those quick revisions on the fly for the parent.
	F3: And I thought that obviously [Facilitator 1] is great at adjusting... [and] in the moment going like “This isn’t working, we should do something else”, so I think that was great. So I think not sticking to the plan too well was really what made the PLAYshop work when we did it.
	F4: … also one of the things I witnessed when I sat in on other [workshops] was the kind of optimism and enthusiasm and energy from a facilitator. So, that really has an impact on how engaged the parents become themselves. and how enthusiastic and energetic they are.
**Separating parents and children**	F1: So, reflection and discussion is an important piece of the adult education experience and so that also needs the kids pulled away
	F2: Umm, so we did allow people to bring their kids. Um, which was good because then we had enough kinda facilitators there to break off into 2 groups. So when we needed to speak to the parents by themselves we could, and then someone would play games with the kids and then we would come back as a big group.
	F3: We did [the workshop]- the ones that I helped out with- we did in 2 elementary school gyms and that's like the perfect amount of space.
	F4: … just really making sure that [the space] is a setting conducive to movement and to physical literacy type game[s] and play.
**Parent response and engagement**	F1: So, the parents were really enthusiastic… They asked questions. …. they were very engaged.
	F4: The parents seemed to receive [the workshop] well. We did have some parents with their kids there and the kids [also] seemed to really be engaged and it was a positive atmosphere.
	F2: I think the actual content of [the workshop] was good, parents seemed to like that. So I wouldn't change necessarily that aspect.
	F4: … and then those key messages… watching some of the other workshops that were facilitated primarily by [Facilitator 1] really the emphasis on those key messages about being playful and fun but also [using the play] in order to develop those skills and the importance behind that.

**Table 4:** Workshop challenges and areas for improvement from the perspective of workshop facilitators

Workshop challenges and areas for improvement	
**Theme**	**Quotes**
**Workshop recruitment and attendance**	F1: Recruitment. It is so difficult getting the parents [to the workshop]. It is very random. So typically when you're recruiting in all these settings you’re going through a third party, like the parent advisory committee person, inviting the parents through their network or the school principal or the sport club technical director. So, you have to rely on somebody to send out the notices to the list. So that broke down in some cases, but not all.
	F3: I think participation was the biggest one… it’s easy enough to put it out there and say this is gonna happen and I think in theory like people want to know this sort of stuff … but like when it comes time to actually deliver the programs a lot of those like “maybes, ya sortas” turn into no-shows.
**Unfavorable spaces**	F1:…outside space is problematic I would suggest that the workshop should be done indoors and mostly because we use a lot of balloons and light things and they blow away. The second thing is the ability to bound your space. It's a bit chaos-y. You need an ability to bound your space
	F4: … ya the classroom was a little broken [up] because of all the chairs and the tables and I remember thinking that it would be better had I pushed like the stuff aside and just created a more open inviting atmosphere.
	F3: I think we tried to do it all together and when you’re- it’s like 7 o’clock after school and kids are expecting to be like playing games and you’re trying to explain things to the parents while the kids are just standing there and watching you do it, you lose a lot of that attention because the parents are now worried about what the kids are doing instead of what you’re explaining to them about.
	F4: ….. when the parents that had small children with them, I think it was harder, it was more difficult for them to (pause) focus and really get the most out of the workshop. ….
**Supporting parents after the workshop**	F1: … I would do a let’s make activity and they would leave with the piece of equipment ……We did do a [simple] handout, …. but a more professional handout and a web resource where they could go to find some simple ideas probably would help.
	F4: [The workshop] is engaging and parents get motivated but then once they leave- what are the chances that it’s sustained that kind of you know learning and positive energy. … so I think some sort of- follow-up, some sort of sustainability type strategy just to prompt that continued behaviour.

## Discussion

We conducted a small, uncontrolled study to assess the preliminary feasibility (limited efficacy and acceptability [7]) of a brief, parent-focused childhood physical literacy intervention. The theory-based PLAYshop aimed to enhance the knowledge, confidence, skills, and resources necessary to support parents to assist their child to develop physical literacy through play. The findings were positive, demonstrating that the intervention was feasible (highly acceptable and easy to implement) and potentially efficacious; however, program recruitment was challenging and areas for improvement in implementation were identified.

The PLAYshop shows promise as an early childhood physical literacy intervention option for building parents’ knowledge and self-efficacy for playing purposefully with their child to develop physical literacy and physical activity. Compared to pre-workshop measures, post-workshop surveys of parents immediately following program delivery showed significantly higher levels of knowledge in key physical literacy variables and confidence in undertaking physical-literacy promoting activities with their child/children. Further, the majority of parents were highly motivated to apply learnings from the PLAYshop and undertake physical literacy activities with their child/children, and parents’ engagement in purposeful play with their child/children at home reportedly increased following participation in a workshop.

Consistent with findings of prior research [3, 25–27], our results suggest that a brief training workshop may positively influence parenting practices with regard to physical literacy. Other studies have shown that the home environment impacts the motor skill development of children [4, 36] and RCTs have further linked parent-focused interventions with improvements in child’s FMS [27] and physical activity [25–27]. This has important implications seeing as physical literacy is a lifelong journey that impacts numerous health outcomes [9]. Parent training and education may provide a viable means of influencing the physical literacy journey early in the life course.

According to our results, the PLAYshop also appeared acceptable from the perspective of those involved. The majority of parents reported that they were “highly satisfied” with the program. Interviews of parents and workshop facilitators highlighted numerous strengths relating to the PLAYshop content and delivery, including its usefulness, convenience and ability to elicit enthusiasm from both parents and children. Further, the workshop brevity, ease of delivery, and low supplies required make it a realistic public health intervention in an era where resources are often scarce and increase its likelihood of successful scale-up [35]. Despite these strengths, several implementation barriers were noted, such as distracting children, difficulty recruiting parents, and unfavorable delivery spaces. Fortunately, these challenges were exposed in this feasibility study and may now be mitigated by intervention adaptations. For example, an additional facilitator to engage children during parental learning might lessen the distraction—a method successfully employed for the HDHK father-child practical sessions [25, 26].

This study has numerous limitations including the small number of participants (largely due to recruitment challenges) and lack of a control group. However, this is common of feasibility studies whose primary purpose is to determine whether future definitive trials of an intervention should take place and if so, what this trial should like [6]. Another limitation is the possibility of selection bias due to the use of self-recruitment methods: parents that were engaged with physical activity may have been more likely to enroll in the study. Future research is needed to determine how to recruit parents who are less engaged with physical activity for a more representative sample. Lastly, this study is limited by the short time period between baseline and follow-up data collection, with post-workshop surveys conducted the day following parent participation in the workshop. Longer term follow-up is needed to determine if the effects seen immediately following intervention delivery are maintained. Strengths of the study included the workshop delivery with real-world partners and in the context in which potential scale-up would occur.

## Conclusion

The PLAYshop appeared feasible—it improved parent knowledge and confidence to promote physical literacy with their child/children, motivated physical literacy promoting practices, and was highly acceptable to the target audience, although recruitment was challenging. A more rigorous assessment of the PLAYshop via a larger trial is now needed to address the limitations of the current study and establish its efficacy. The findings from this study will inform adaptations to improve intervention implementation and outcomes in such a full-scale efficacy trial. In terms of the intervention impact, follow-up support for parents was recommended, and in terms of workshop implementation, an additional leader was encouraged. Enhanced recruitment efforts will be needed in a future trial, in particular more direct parent recruitment by the research team.

This study addresses an important research gap and is a valuable preliminary step in the development, testing, and delivery of a scalable parent-focused intervention to promote childhood physical literacy. It contributes to the broader physical literacy movement that strives for children to have “the motivation, confidence, knowledge, skills, and fitness necessary to enjoy a physically active lifestyle” [20].

## Data Availability

The datasets used and/or analyzed during the current study are available from the corresponding author on reasonable request.

## Abbreviations

IPLA: International Physical Literacy Association
HDHK: Healthy Dads Healthy Kids
DADEE: Dads and Daughters Exercising and Empowered
FMS: Fundamental movement skills
RCT: Randomized controlled trial
BCW: Behavior change wheel
